# Exploring a New Therapy for Diabetic Polyneuropathy – The Application of Stem Cell Transplantation

**DOI:** 10.3389/fendo.2014.00045

**Published:** 2014-04-09

**Authors:** Hiroki Mizukami, Soroku Yagihashi

**Affiliations:** ^1^Department of Pathology and Molecular Medicine, Hirosaki University Graduate School of Medicine, Hirosaki, Japan

**Keywords:** diabetic neuropathy, cell therapy, stem cell, adipose tissue-derived mesenchymal stem cell

## Abstract

Diabetic polyneuropathy (DPN) is the most common complication that emerges early in diabetic patients. Intervention with strict blood glucose control or treatment with aldose reductase inhibitor is reported to be effective in early stages of DPN. Curative treatment for overt or symptomatic DPN, however, has not been established, thus requiring much effort to explore a new therapy. Recent preclinical studies on the use of gene or cell therapy have provided promising results in the treatment of DPN. Of particular interest, induced pluripotent stem cells are introduced. In these studies, restoration of DPN was proposed to be attributed to either neurotrophic factors released from transplanted stem cells or differentiation of stem cells to substitute the damaged peripheral nerve. There are still several problems, however, that remain to be overcome, such as perturbed function, fragility, or limited survival of transplanted cells in diabetes milieu and risk for malignant transformation of transplanted cells. Questions, which cell is the most appropriate as the source for cell therapy, or which site is the best for transplantation to obtain the most effective results, remain to be answered. In this communication, we overview the current status of preclinical studies on the cell therapy for DPN and discuss the future prospect.

## Introduction

Diabetic polyneuropathy (DPN) is an early and frequent complication of diabetes. Before symptoms become evident, most diabetic patients exhibit more or less abnormal peripheral nerve function such as prolonged F wave latency or reduced nerve conduction velocity of sural and tibial nerves, indicative of early metabolic interference to nerve integrity (1). Population of small fibers in the skin observed by confocal laser scan microscopy is also found to be dramatically reduced in patients with impaired glucose tolerance, pre-diabetic stage of type 2 diabetic patients (2). It is thus likely that there is almost no patient who does not suffer from incipient progression of nerve damage in the presence of diabetes. Current consensus on the diagnosis of DPN depends on clinical and/or electrophysiological abnormalities in peripheral nerve after exclusion of the causes other than diabetes. Distal dominant polyneuropathy is the most frequent type of diabetic neuropathy, and further divided into symmetric sensory dominant neuropathy and autonomic neuropathy. Underlying pathology of diabetic neuropathy is characterized by a progressive distal axonal degeneration, axonal loss, and demyelination (3, 4) accompanied by microvascular changes, which exhibit typical features of microangiopathy with thickening of basement membrane and luminal occlusion (5–7). Under such circumstances, peripheral nerve tissues undergo ischemia and poor supply of nerve nutrients as well as nerve trophic factors, thus contributing to development of neuropathy. In addition, local depletion of neurotrophic factors such as nerve growth factor (NGF), insulin-like growth factor (IGF)-I, and vascular endothelial growth factor (VEGF) may attribute to the distal pre-dominant nerve pathology.

Patients with such pathology complain intractable pain, insomnia, and depression. In advanced stage, contracted foot may be amputated in the absence of perception by the reason for non-curable diabetic gangrene and local infection. Thus, the neuropathy impairs quality of life, resulting in premature death of the patients.

While a number of reports disclosed that intervention with tight blood glucose control, treatment with aldose reductase inhibitor or alpha-lipoic acid successfully suppressed the progression of neuropathy (8–10), there is no established curable treatment in progressive stage. Notwithstanding, there have been continuous efforts to develop new means to combat with this serious disorder. One of innovative preclinical studies may be depicted as application of gene therapy or cell therapy to experimental diabetic neuropathy in animal models (11). Among those, there are several reports that exhibited promising results. As a source of cell therapy, embryonic stem cell (ES cell), inducible pleuriopotent stem cell (iPS cell), bone marrow-derived mononuclear cell (BMMNC), mesenchymal stem cells (MSC), and others as well may be listed (Table 1). ES cell is excluded in this group because of the ethical problems and a risk of tumor formation. We will discuss in this communication on the effects of endothelial progenitor cells, MSC, and induced pluripotent stem (iPS) cells on experimental diabetic neuropathy.

**Table 1:** **Source of cell therapy for experimental diabetic neuropathy**.

Name of stem cells	Advantages	Weak points
Cord blood-derived endothelial progenitor cell (CB-EPC) (12)	Efficient utilization of cords that is unused	No application to autologous transplantation
Bone marrow-derived endothelial progenitor cells (BM-EPC) (13)	Transdifferentiation into local intrinsic endothelial cells	*Ex vivo* expansion
		Functional loss in diabetes
Peripheral blood mononuclear cell (PBMNC) (14)	Low invasiveness	Functional loss in diabetes
	Application to autologous transplantation	
Bone marrow mononuclear cell (BMMNC) (14–16)	Application to autologous transplantation	Functional loss in diabetes
Inducible pluripotent stem cells (iPSC) (17)	Utilization of adult somatic cells	Gene introduction using virus vector
		Tumor formation
Bone marrow-derived mesenchymal stem cell (BMMSC) (18–20)	Low invasiveness	*Ex vivo* expansion
		Tumor formation
		Functional loss in diabetes
		Short effective period
Adipose tissue-derived mesenchymal stem cell (ASC)	Low invasiveness	Short effective period
	No *ex vivo* expansion	Functional loss in diabetes

## Endothelial Progenitor Cells

Naruse et al. examined the effects of cord blood-derived endothelial progenitor cells (CB-EPC) on neuropathic changes in streptozotocin (STZ)-induced diabetic rats (12). In this study, 1 × 10^6^ number of CB-EPC were transplanted into quadriceps muscle, biceps femoris muscle, and soleus muscle in nude rats with 8-week diabetic duration. Four weeks after transplantation, treated group showed improvement of motor nerve conduction velocity (MNCV) and sciatic nerve blood flow (SNBF) only in transplanted limb compared to non-treated side. They interpreted that the recovery of nerve function was accounted for by improved SNBF related to increased vascular density in the treated leg muscles. Jeong et al. applied bone marrow-derived endothelial progenitor cells (BM-EPC) to neuropathy in STZ-induced diabetic mice (13). BM-EPC transplantation improved not only MNCV and SNBF, but also increased the density of vasa nervorum in the sciatic nerve. To our surprise, transplanted BM-EPC migrated along the vessel wall of sciatic nerve and fused with endothelial cells, intrinsic to the host mouse. Upregulated expression of mRNA or proteins of VEGF, basic fibroblast growth factor (bFGF), brain-derived neurotrophic factor (BDNF), and stromal cell-derived factor 1a was also demonstrated in the sciatic nerve. From these results, it is likely that EPC transplantation is beneficial for treatment of diabetic neuropathy thorough augmented expression of neurotrophic factors and improvement of vascular function perhaps based on increased blood vessels in part associated with newly built endothelial cells derived from EPC. It is yet to be clear, however, in these studies, to which extent transplanted cells survive or to what transplanted cells differentiate into. Longer observation should be essential to answer to this question.

## Peripheral Blood Mononuclear Cell and Bone Marrow Mononuclear Cell

Mononuclear cells which contain various kinds of cell lineages, such as hematopoietic cells, fibroblasts, osteoblasts, and myogenic cells as well as endothelial lineage, can work beneficially in various settings (21). The effects of peripheral blood mononuclear cell (PBMNC) and BMMNC on neuropathy in STZ-induced diabetic rats were examined by Hasegawa et al. (14). After 4 weeks of diabetes, 6 × 10^7^ PBMNC or 1 × 10^6^ BMMNC were transplanted into the hindlimb muscle. Four weeks after transplantation, both MNCV and SNBF on transplanted limb were significantly improved while those in non-transplanted limb remained low. The effects of either PBMNC or BMMNC were set off when neutralizing antibody to VEGF was pre-treated. From these results, the therapeutic effects of PBMNC and BMMNC may mainly be attributed to the improvement of VEGF-mediated vascular function, and share common with the effects of EPC. In contrast to EPC, however, microvascular density in the sciatic nerve was not influenced by MNC therapy. More recent study conducted by Kim et al. demonstrated robust improvement of neuropathy in STZ-induced diabetic rats transplanted with 5 × 10^5^ BMMNC along the sciatic nerve (15). Indeed, transplantation of BMMNC ameliorated MNCV, sensory nerve conduction velocity (SNCV), and SNBF as well as perineurial microvessel density around the sciatic nerve. They further found migration of transplanted BMMNC into the sciatic nerve along the endoneurial vessels although no obvious fusion of MNC with endothelial cells was identified. Concurrently, mRNA expression of neurotrophic factors such as VEGF, bFGF, IGF-I, and nitric oxide synthase-3 in transplanted site was significantly restored compared to the other site. More recently, a study conducted by Naruse et al. showed improvement of hyperalgesia and mechanical allodynia in STZ-induced diabetic rats transplanted with autologous BMMNC into gastrocunemius muscle (16). They assumed that the local recovery of microcirculation in the muscle was the main cause for the therapeutic efficacy of autologous BMMC. Intriguingly, but requiring caution for the interpretation, intraepidermal nerve fiber density (IENFD) was not affected in their study. It is thus likely that MNC ameliorates neuropathy mainly mediated by vascular function, but it remains unclear whether the functional recovery links with structural repair.

## Induced Pluripotent Stem cell

Induced pluripotent stem cells are stem cells induced artificially from mature somatic cells by insertion of four genes (*oct3/4, sox2, klf4, c-myc*) using a vector of retrovirus (22). There is a possibility of tumor formation from iPS cells because *c-myc* is one of the potent oncogenes. Recently, *L-myc* or *glis-1* is alternatively applied to the induction of iPS cells as being safer than *c-myc* (23, 24). Moreover, three candidate genes were identified as non-tumorigenic iPS cell (25). According to the efforts to improve the quality of iPS cells, transplantation therapy of iPS cells is expected to be safer than before.

There is one publication that introduced beneficial effects of iPS cells on experimental diabetic neuropathy (17). The authors produced iPS cells from aged mouse to develop into neural crest cells, which in turn possessed a potency to differentiate into the constituents of peripheral nerve tissues including neurons, Schwann cells and vascular smooth muscle cells. The differentiated iPS cells were transplanted into hindlimb of STZ-induced diabetic mice with 16-week diabetic duration. Four weeks after transplantation, NCVs, nerve population in the footpad (IENFD), perception impairments to thermal stimuli were significantly improved. In these animals, the improvement of neuropathic changes was associated with recovery of sciatic nerve and plantar skin blood flow and increased capillary density in the muscle. They further found increased protein expressions of neurotrophic factors such as NGF and VEGF only in the sciatic nerve, but not in the DRG. It was thus indicated that the effects of transplanted cells were only local not to the distant tissues. In this study, the investigators exhibited the illustrations of differentiation of implanted iPS cells to vascular smooth muscle cells or Schwann cell-like cells in each counterpart of the recipients. Nevertheless, the mechanism of how iPS therapy influenced on neuropathy may be more or less similar to those in other kinds of stem cell therapy, which induces or enhances local release of neurotrophic factors. Several questions still remain as to how long the implanted cells can survive at the local site, or to which extent there is a risk for the tumor formation. If we can overcome these obstacles, iPS cell therapy could be the most promising for the advanced stage of neuropathy because ample amount of iPS cells will be available for transplantation by its easy procurement from mature somatic cells.

## Mesenchymal Stem Cell

Mesenchymal stem cells are a group of stem cells, which are contained in various adult tissues including bone marrow, adipose tissue, and spinal ligaments (26–28). These cells express CD44, CD90, or CD105 as surface markers. Two kinds of MSC are representative; one purified from bone marrow (bone marrow-derived MSC; BMSC) and another from adipose tissue (adipose tissue-derived MSC: ASC). Since ASC seems to be migrated BMSC into the adipose tissue, there is no marked phenotypic difference between ASC and BMSC (29, 30). It is of note that pericytes that surround small capillaries express cell surface phenotype (CD44, CD90, and CD105) similar to those possessed by MSC that has multipotential differentiation into bone or cartilage. Thus, a part of pericytes are considered to be a precursor of MSC (31). In fact, the infusion of MSC into blood vessels *in vivo* resulted in localization of MSC surrounding vessels that differentiated into pericyte (31).

Several reports disclosed the effects of MSC therapy on experimental diabetic neuropathy. Shibata et al. transplanted 1 × 10^6^ BMSC into femoral and gastrocunemius muscle of STZ-induced diabetic rats with 8 weeks duration (18). BMSC transplantation significantly improved MNCV, SNBF and increased density of small vessels in the muscle in the transplanted limbs 4 weeks after the transplantation. They found increased mRNA expressions of VEGF and bFGF, and neurofilament protein expression in the transplanted site. Myelinated fiber morphometry revealed restored axonal circularity in BMSC-implanted limb of diabetic rats, but no changes in myelin area in those animals suggesting limited effects on myelination. Kim et al. also transplanted 1 × 10^6^ BMSC in the muscles along the sciatic nerve in STZ-induced diabetic mice with 12-week diabetic duration (19). Two weeks later, they found significant improvement of mRNA expression of neurotrophic factors such as NT-3 and NGF. Contrary to the expectation, however, the effects of BMSC diminished 4 weeks after the transplantation. From the above two reports, the effective period of the BMSC transplantation therapy was found to be only 2–4 weeks after transplantation regardless of the animal species or duration of diabetes. In their studies, not autologous, but allogenic BMSC was used for transplantation. Thus, the dwindling of the efficacy in transplanted animals may be ascribed to the removal of the cells by the immune system of recipient. The limitation of allogenic MSC therapy may also be related to the gradual decrease in released neurotrophic factors from transplanted cells that may sustain only 4 weeks or so after transplantation. Another issue that should be overcome for the MSC therapy is to avoid the risk of tumor formation. In fact, Jeong et al. found frequent tumor formation in STZ-induced diabetic mice transplanted with BMSC (20). In this study, 4–8 weeks after the transplantation, sarcoma with muscle phenotype was identified in the transplanted site in 46% of treated animals. The high frequency of tumor formation was accounted for by frequent chromosomal mutation elicited by repeated passages of BMSC (only four passages) in the cell culture system. It may thus be conceivable that fresh BMSC without any passage in cell culture should be applied to cell therapy to prevent tumor formation.

## Advantage of ASC in Clinical Application

Adipose tissue-derived mesenchymal stem cell has unique advantage over the other kinds of MSC that do not have, represented by the fact that, (1) less invasive procedure is required for its isolation, (2) ASC locates in mature adipose tissue, (3) there is no need for *ex vivo* culture after isolation, (4) automatic cell sorter is available on the market (29, 32), and (5) ASC is less immunogenic. By the reason of (1–5), autologous cell transplantation of ASC is feasible as a source of cell therapy. In case of BMSC, *ex vivo* culture is indispensable because the volume of bone marrow blood available for one time is restricted. Litter of the fatty tissue is allowed to collect by lipoaspiration technique, enabling primarily isolated ASC for transplantation. As mentioned in the previous section, there is a risk that *ex vivo* culture may cause chromosomal abnormality of MSC. Thus, ASC seems to be superior for clinical application compared to other kinds of MSC. Indeed, ASC is already clinically applied to cardiovascular diseases like acute myocardial infarction, wound healing of skin ulcer, or plastic surgery such as mammoplasty as well as facial repair. Good news is that safety of the ASC therapy has already been confirmed (33).

## Mechanism of Tissue Repair in ASC Therapy

Adipose tissue-derived mesenchymal stem cell can induce tissue repair through several potential mechanisms mediated by (1) neurotrophic factors released from transplanted ASCs, (2) differentiation and engraftment of ASCs in the target organs, (3) immunosuppressive effects of ASCs. It may be conceivable that the effects of ASCs are largely based on released neurotrophic factors. ASCs produce a variety of neurotrophic factors such as epidermal growth factor, transforming growth factor-β, VEGF, bFGF, hepatocyte growth factor, IGF-I, and BDNF. Of these factors, the expression of VEGF, bFGF, IGF-I, and BDNF was shown to be decreased in peripheral nerve tissues of experimental diabetic neuropathy. ASCs transplantation can restore the expression of these neurotrophic factors, which in turn ameliorates neuropathy. The underlying process may be similar to the substitution of these neurotrophic factors. As already alluded, most transplanted stem cells cannot survive in transplanted site for a long time (less than 1 month). It may therefore be difficult to expect differentiation and engraftment of ASCs in transplanted site like other stem cells. Immunosuppressive effect of ASC may also be expected by the recent experimental findings, which showed suppressive effects of ASCs on inflammatory reactions in the animal models of fulminant hepatitis, ulcerative colitis, and Crohn’s disease (34, 35). Since inflammatory processes contribute to the development of diabetic neuropathy, the effects of ASC may in part be ascribed to the counteraction against proinflammatory reactions. However, this issue should be confirmed by future investigations.

In our own experience, we found significant recovery of nerve conduction delay in STZ-induced diabetic rats 2 weeks after ASC transplantation. Concurrently, the atrophy of the ganglion cells in DRG was improved. mRNA expression of the neurotrophic factors such as VEGF in soleus muscle at the transplanted site was increased while contralateral limb was not affected. Histological evaluation could not identify differentiation of ASC to either neuronal cells or Schwann cells, nor increase in capillary density in the muscles 4 weeks after the transplantation. Summarizing these results, ASC can improve the peripheral nerve function in diabetic neuropathy through the increase in the local expression of neurotrophic factors.

## Effects of Stem Cells on Diabetic Skin Ulcer

Diabetic foot ulcer is common but serious complications of diabetes frequently associated with DPN and suitable target for cell therapy. Previous reports exhibiting the effectiveness of diversity of stem cells including MSC, EPC, and previous literature disclosed the beneficial effects of BM–BMMC on the wound repair in experimental diabetic animal model (36). The mechanisms that operate in those models are more or less similar to those alluded in the case of the DPN involving replenishment of growth factor production, suppression of the sustained inflammation, and improvement of blood supply by neovascularization. Small population of transplanted stem cells could differentiate into endothelial cells in transplanted sites. It is less likely, however, that this mechanism in fact is a major process for the recovery because of a paucity of such differentiated cells.

## Diabetes and Stem Cell Dysfunction

Hyperglycemic state may affect stem cell function. There are several publications that showed functional impairment of MSCs, EPC, and BMMC (37–39). They underwent reduced proliferative activity (38), impaired fibrinolytic activity (40), poor differentiation into endothelial cells or osteocytes (41), but untoward differentiation into adipocyte (42), and induction of apoptosis (38). EPC procured from diabetic patients suffers from low yield dependent on high HbA1c and low proliferation activity with impaired tubule formation (43, 44). The dysfunction of EPC was proposed to be relevant to insulin resistance, cellular senescence, or other glucose-dependent cell damage like advanced glycation end products (AGE) formation as well as alterations of its receptor (RAGE) (38, 41, 45).

## Future Perspective of Cell Therapy

**Figure 1:**
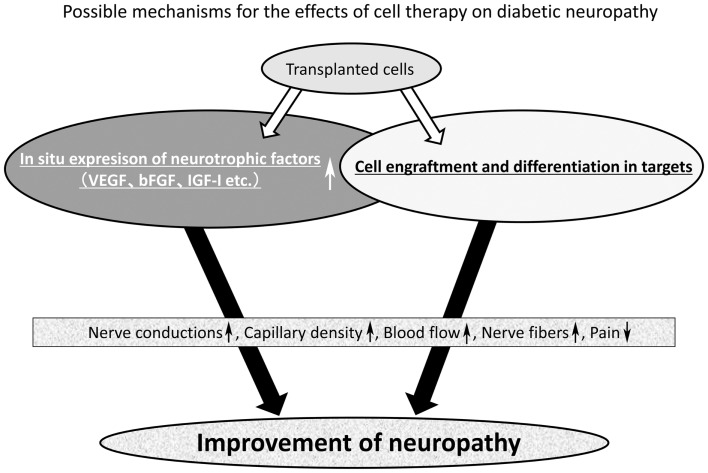
**Mechanism of the effect of stem cell transplantation on diabetic neuropathy**. The mechanism of nerve repair by stem cell transplantation may be divided into two main pathways. One is the enhancement of local expression of neurotrophic factors and another is the cell engraftment and differentiation into tissue constituents of large tissues. Most stem cells can improve the local expression of neurotrophic factors, but from the literature only two kinds of stem cells (bone marrow-derived endothelial progenitor cells and iPS cell) were found to achieve cell engraftment and differentiation in the target tissues. Collectively, stem cells can improve motor and sensory nerve conduction velocity (MNCV and SNCV), sciatic nerve blood flow (SNBF), capillary density, intraepidermal nerve fiber density (IENFD), hyperalgesia, and mechanical allodynia.

As shown in Figure 1, stem cells can improve diabetic neuropathy through two main pathways. One is the enhancement of the expression of local neurotrophic factors, and another is the engraftment and differentiation into tissue constituents in target tissues (Figure 1). Cell therapy may be applicable not only to acute serious illnesses like myocardial infarction, but also to chronic disorders represented by diabetic neuropathy. There is a great expectation of the development of cell therapy effective for advanced stage of neuropathy or severe painful neuropathy in diabetes because we lack effective treatment except for palliative observation of the patients.

Despite numerous promising results from hitherto published preclinical studies, there appear to be many obstacles to be overcome for clinical applications. Those are: (1) limited survival of transplanted cells, (2) risk of tumor formation, (3) cost and effect relationship, (4) undetermined site of transplantation for safety and effectiveness, (5) choice of the most suitable stem cells tailored for a given patient, (6) impaired potency of stem cells derived from diabetic patients, and finally (7) unestablished clinical endpoints for the efficacy of cell therapy. Thus, much more information is needed before considering clinical application.

## Conflict of Interest Statement

The authors declare that the research was conducted in the absence of any commercial or financial relationships that could be construed as a potential conflict of interest.
